# Influence of limit-feeding duration on growth performance and carcass traits of finishing steers

**DOI:** 10.1093/tas/txag139

**Published:** 2026-09-12

**Authors:** Justin J Delver, Federico Podversich, Ana C B Menezes, Warren C Rusche, Dale A Blasi, Logan R Thompson, K C Olson, John P Ritten, Zachary K F Smith

**Affiliations:** Department of Animal Science, South Dakota State University, Brookings, SD 57007, United States; Department of Animal Science, South Dakota State University, Brookings, SD 57007, United States; Department of Animal Science, South Dakota State University, Brookings, SD 57007, United States; Department of Animal Science, South Dakota State University, Brookings, SD 57007, United States; Department of Animal Sciences and Industry, Kansas State University, Manhattan, KS 66506, United States; Department of Animal Sciences and Industry, Kansas State University, Manhattan, KS 66506, United States; Department of Animal Sciences and Industry, Kansas State University, Manhattan, KS 66506, United States; CSU AgNext, Department of Animal Sciences, Colorado State University, Fort Collins, CO 80523, United States; Department of Animal Science, South Dakota State University, Brookings, SD 57007, United States

**Keywords:** beef, carcass, feed restriction, growth

## Abstract

Beef cattle are commonly limit-fed during the backgrounding period to improve feed efficiency and control fat deposition, but the effects of limit feeding duration in the finishing period remains unclear. The objective of this study was to determine the effect of limit feeding yearling beef steers for 56 or 86 d before offering ad libitum feed for the remainder of the 141-d finishing period on growth performance and carcass traits. Black Angus steers (*n* = 194; initial shrunk body weight [SBW] = 410 ± 17.9 kg) were allocated into 24 pens (8 steers/pen) and assigned to one of three treatments in a randomized complete block design: ad libitum intake for the entire finishing period (*ADLIB*), 92% of ad libitum intake for 56 d followed by ad libitum intake for the remainder (*RES56*), and 92% of ad libitum intake for 86 d then ad libitum intake for the remainder (*RES86*). Feed restrictions began on d 22 following diet adaptation with RES56 and RES86 pens being offered 92% of the ADLIB pen within their block’s daily feed offering. Data were analyzed using the MIXED and GLIMMIX procedure of SAS 9.4 with treatment and block as the fixed and random effects, respectively and d 22 SBW as a covariate. From d 1 to 21 dry matter intake (DMI), average daily gain (ADG), and gain to feed (G:F) did not differ (*P* ≥ 0.17). From d 22 to 107, G:F tended to differ (*P* = 0.10), but ADLIB had a greater DMI than RES56 and RES86 (*P* < 0.01). From d 78 to 107 RES56 were allowed ad libitum feed intake while RES86 were still restricted. The d 108 SBW tended to differ (*P* = 0.09), and DMI was least in the RES86 group (*P* < 0.01). The RES56 and RES86 had the greatest ADG and G:F during this period (*P* < 0.01). From d 108 to 140, all cattle had ad libitum access to feed, and DMI did not differ (*P* ≥ 0.15). The RES86 ADG was greater than ADLIB but not differ from RES56 during this period (*P* = 0.04). Cumulatively, final SBW and ADG did not differ (*P* ≥ 0.71), but the ADLIB group had increased DMI and the least G:F (*P* ≤ 0.04). Ribeye area and HCW did not differ (*P* ≥ 0.27), but rib fat thickness and marbling were greater for ADLIB (*P* ≤ 0.02). Limit feeding yearling beef steers for a portion of the finishing period before resuming ad libitum feed intake reduced cumulative DMI and improved G:F without affecting ADG, final shrunk BW, or HCW; but decreased subcutaneous and marbling fat. Extending days on feed may overcome fatness deficits achieving heavier HCW at similar empty body fat for limit fed cattle.

## Teaser

Limit feeding yearling beef steers for a portion of the finishing period reduced feed intake without affecting growth performance, but reduced carcass fatness and quality grade.

## Introduction

The use of limit feeding in feedlot cattle is generally applied in the growing phase through programmed feeding of high-concentrate diets (Sip and Pritchard 1991; Galyean et al. 1999; Galyean and Hales 2023), the use of slick bunk management (Pritchard and Bruns 2003), and to help mitigate heat stress (Holt et al. 2000). Limit feeding during the finishing phase is primarily done through slick bunk management and to improve feed efficiency but may result in decreased gain and final body weights if cattle are not fed for additional days (Murphy and Loerch 1994; Loerch and Fluharty 1998; Galyean and Hales 2023). The goal of the finishing period is to maximize carcass growth and quality, so limit feeding is often used for a portion of the finishing period to help capitalize on compensatory gain without slowing growth (Galyean et al. 1999).

Determining an optimal duration and degree of restriction is dependent on sufficient severity to elicit a compensatory growth response while avoiding unrecoverable performance losses. Feed restrictions of 5% to 15% of ad libitum intake generally improve feed efficiency, while minimizing risk of such losses (Hicks et al. 1990; Sainz et al. 1995; Galyean et al. 1999). Hicks et al. (1990) characterized a 15% restriction in heifers as severe due to a 7% reduction in gain, while an 11% restriction improved feed efficiency without a significant reduction in gain. Similarly, Plegge (1986) limit-fed steers by 4 and 8% of ad libitum intake and observed improvements in feed efficiency of 2.2% and 4.7%, respectively. This suggests that an 8% restriction represents a practically applicable target that improves feed efficiency without compromising overall live performance.

Limit feeding for a portion of the finishing period can yield similar final live body weights to ad libitum fed cattle, but with reduced cumulative feed intake. This improved feed efficiency is partially due to reducing the energetic costs of maintenance through reductions in gastro-intestinal tract and liver size (Sainz and Bentley 1997). Cattle may also be more efficient due to leaner carcasses and composition of gain, although this can negatively impact carcass quality (Hicks et al. 1990). Steers that were programmed to gain 1.13 kg/d for 70 d had greater subsequent gains in the realimentation period than cattle restricted for only 32 or 47 days (Knoblich et al. 1997). However, to completely recover lost carcass quality, cattle may require up to 100 days of realimentation after a stepwise increase in programmed gain (Loerch and Fluharty 1998). To optimize limit feeding within the finishing period, restriction duration and realimentation duration must be carefully balanced to capture compensatory gain without extending days on feed or sacrificing carcass quality.

The objective of this experiment was to determine the effects of limit feeding yearling beef steers 92% of ad libitum intake for 56 or 86 d, followed by an ad libitum realimentation period of 64 or 34 d, respectively, on growth performance and carcass traits.

## Materials and methods

This study was conducted at the Southeast Research Farm in Beresford, South Dakota, between September 2023 and February 2024. The animal care and handling procedures used in this study were approved by the South Dakota State University Animal Care and Use Committee (2306-052E).

### Animals, experimental design, and dietary treatments

Two hundred and thirty-six yearling Black Angus steers (arrival BW = 431 ± 17.2 kg) were procured from an auction barn in South Dakota (Kimball Livestock Exchange, LLC; Kimball, SD) and were transported to the Southeast Research Farm (Beresford, SD) on September 19, 2023 (d 6). Upon arrival cattle were group housed (∼ 10 steers/pen) and offered their step 1 diet at 1.75% of BW. On d 3 all cattle were weighed for allotment, administered a unique visual and radio-frequency identification (RFID) ear tag, vaccinated against infectious bovine rhinotracheitis virus, parainfluenza3 virus, bovine respiratory syncytial virus; bovine viral diarrhea Types 1a, 1b, and 2 virus, and *Mannheimia haemoplytica* type A1 (Bovishield Gold Oneshot, Zoetis, Parsippany, NJ) and *Clostridium chauvoei, Cl. Novui, Cl. perfringens* Types B, C, and D, and *Histophilus somni* (Ultrabac 7/Somubac, Zoetis), as well as administered a pour-on anthelmintic (Cydectin, Elanco Animal Health, Indianapolis, IN) according to label directions. On d 1, 194 of the steers were allotted to treatment pens (8 steers/pen; 24 pens) using the previous weights and were reweighed for d 1 BW. To produce a similar BW distribution within pens, cattle were sorted by their d 3 BW and assigned using a staggered rotation to 1 of 8 replicates (1–8; 2–8, 1; 3–8, 1–2; 4–8, 1–3; 5–8, 1–4; 6–8, 1–5; 7–8, 1–6; 8, 1–7). Cattle were then sorted by replicate and BW and assigned to 1 of 3 treatments using a staggered rotation (1, 2, 3; 2, 3, 1; 3, 2, 1). This generated 24 pens (8 steers/pen; 8 pens/treatment) in a randomized complete block design where they were blocked by location within the feedyard. The treatments were:

1. Offered *ad libitum* feed the entire feeding period (*ADLIB*; *n* = 64)
2. Offered 92% of *ad libitum* feed from d 22 to d 77 (56 d) then offered *ad libitum* feed from d 78 to d 141 (*RES56*; *n* = 64)
3. Offered 92% of *ad libitum* feed from d 22 to d 107 (86 d) then offered *ad libitum* feed from d 108 to d 141 (*RES86*; *n* = 64)

The RES56 and RES86 restrictions were determined by offering 92% of the ADLIB group within their respective blocks. Table 1 presents a timeline of the feeding management for the three treatments. The ADLIB group, as well as the RES56 and RES86 groups during their respective ad libitum periods, were managed to have a thin layer of feed remaining in the bunk the following morning. Weigh backs were collected, weighed, and thrown away weekly and that feed was subtracted from their feed deliveries for that week to determine feed intake. Feed restrictions began on d 22 after the cattle had been stepped up to their final diet. The cattle were stepped up to their final diet over 4 steps lasting 7 days with the final step being the finishing diet fed during the experiment. Also, on d 22 cattle were reweighed and implanted with 200 mg trenbolone acetate and 20 mg estradiol - 17β (Revalor 200, Merck Animal Health, Rahway, NJ). Restricted cattle were returned to ad libitum feed intake by offering 4% more feed the day the restriction ended, then following normal bunk calls to target a thin layer of feed leftover in the morning. Table 2 presents the bunk calls for the total, restriction, and realimentation periods for the three treatments.

**Table 1:** Timeline of dietary treatments relative to study initiation.

Treatments	d 1 to 21	d 22 to 77	d 78 to 107	d 108 to 141
**ADLIB**	Step-up	Ad libitum	Ad libitum	Ad libitum
**RES56**	Step-up	92% restriction	Ad libitum	Ad libitum
**RES86**	Step-up	92% restriction	92% restriction	Ad libitum
**Weigh days**	d 1, d 22	d 50, d 78	d 108	d 141

Cattle were fed ad libitum for the entire feeding period (ADLIB), restricted to 92% of ad libitum for 56 d (d 22 to 77) then offered ad libitum for the remainder (RES56), or restricted to 92% of ad libitum for 86 d (d 22 to 107) then offered ad libitum for the remainder (RES86).

**Table 2:** Proportion of bunk calls that were a 0 (no feed left in bunk in the morning), 0.5 (thin layer of feed that does not cover the bottom of the bunk leftover in the morning), or ≥ 1 (layer of feed that covers the bottom of the bunk in the morning) for cattle fed ad libitum for the entire feeding period (ADLIB), restricted to 92% of ad libitum for 56 d (d 22 to 77) then offered ad libitum for the remainder (RES56), or restricted to 92% of ad libitum for 86 d (d 22 to 107) then offered ad libitum for the remainder (RES86) during the cumulative, restriction, and realimentation periods.

	ADLIB	RES56	RES86
**Cumulative**			
**0**	59.7%	76.1%	88.1%
** 0.5**	28.6%	17.3%	8.4%
** ≥1**	11.7%	6.6%	3.5%
**Restriction**			
** 0**	51.4%	95.4%	93.3%
** 0.5**	36.8%	4.2%	5.7%
** ≥1**	11.8%	0.4%	1.0%
**Realimentation**			
** 0**	50.7%	50.4%	65.9%
** 0.5**	28.8%	35.3%	21.6%
** ≥1**	20.5%	14.3%	12.5%

Steers were housed in open lot dirt pens (35 m × 14 m) with 6 m concrete bunks and 3 m concrete aprons. All pens were equipped with heated continuous flow water troughs that were shared between two pens. Individual ingredient samples were collected weekly and analyzed for dry matter (DM). Weekly ingredient samples were composited by month and analyzed for N (method no. 968.06 (AOAC2016); Rapid Max N Exceed, Elementar, Mt. Laurel, NJ) and ash (method no. 942.05; AOAC2012). Dry rolled corn was not analyzed for neutral detergent fiber (NDF) or acid detergent fiber (ADF) but assumed a NDF and ADF content of 9% and 3%, respectively; while sorghum silage, grass hay, and modified distillers grain plus solubles (MDGS) were analyzed for NDF and ADF according to Goering and Soest (1970) with the addition of sodium sulfite and alpha amylase to control for protein contamination. Tabular ether extract (EE) values were used for all ingredients except MDGS, which were analyzed using an Ankom Fat Extractor (XT10: Ankom Technology, Macedon, NY). Actual diet formulation based upon weekly DM determination and feed batching records with tabular energy values according to Preston (2017) are presented in Table 3. Steers were fed once daily at 0800 with feed mixed in a commercial mixer wagon (6.1 m^3^; Reel Auggie 3120, Kuhn North America, Inc., Brodhead, WI) with a scale resolution of 0.91 kg.

**Table 3:** Ingredient and nutrient composition of diets fed to yearling Black Angus beef steers.

	d 1 to 7	d 8 to 14	d 15 to 77	d 78 to 141
**Ingredients, % DM**				
** Dry Rolled Corn**	49.1	57.6	68.5	69.8
** MDGS^a^**	16.0	18.1	16.6	16.5
** Sorghum Silage**	20.8	20.3	10.8	9.6
** Grass Hay**	10.0	–	–	–
** Suspended Supplement^b^**	4.1	4.0	4.1	4.1
**Nutrient Composition, Dry Matter Basis**				
** Dry Matter, % As Fed**	56.8	58.5	65.8	65.1
** Crude Protein, %**	13.0	13.2	12.9	12.7
** Neutral Detergent Fiber, %**	26.7	21.2	17.0	16.2
** Acid Detergent Fiber, %**	13.3	9.4	7.6	8.6
** Ash, %**	6.1	5.7	5.2	5.3
** Ether Extract, %**	4.7	5.0	5.0	5.1
** Net Energy for Maintenance, Mcal/kg**	1.87	1.97	2.06	2.07
** Net Energy for Gain, Mcal/kg**	1.19	1.29	1.37	1.38
** Monensin, mg/kg**	33.7	32.7	33.7	34.1

a Modified Distillers’ Grains plus Solubles.

b Suspended supplement contained (DM basis): 27% crude protein, 22.25% non-protein nitrogen, 0.80 Mcal/kg net energy for maintenance, 0.54 Mcal/kg net energy for gain, 0.09% ether extract, 16.92% Ca, 0.4% *P*, 1.54% K, 0.24% Mg, 7.92% NaCl, 3.31% Na, 0.43% S, 4.62 ppm Co, 250 ppm Cu, 40 ppm I, 50 ppm EDDI, 244.93 ppm Fe, 500 ppm Mn, 4 ppm Se, 2253.85 ppm Zn, 79380 IU/kg vitamin A, 19845 IU/kg vitamin D3, 551 IU/kg vitamin E, 827.7 mg/kg monensin sodium.

### Growth performance

All steers were weighed individually at processing, and on days 1, 22, 50, 78, 108, and 141. Steers were weighed in a hydraulic squeeze chute mounted on top of load cells (scale readability ± 0.45 kg) prior to morning feeding. All BW measurements were pencil shrunk 4% to account for gastrointestinal fill (SBW). Initial SBW was calculated as the average of the September 22, 2023 and September 25, 2023 BW shrunk 4%. Dry matter intake (DMI) as a percentage of SBW and metabolic body weight (MBW) were calculated as the average DMI of the feeding period divided by the median shrunk body weight (MeBW) and MBW of the feeding period, respectively. The MBW was calculated as the MeBW to the power of 0.75. Carcass adjusted final SBW was calculated as hot carcass weight (HCW)/0.625. Average daily gain (ADG) was calculated as the difference between final SBW and initial SBW divided by days on feed (DOF) for each weigh period and feed conversion efficiency (G:F) was calculated from ADG/DMI. Cumulative growth responses were determined from d 1 to d 141.

### Carcass characteristics

Steers were fed until visually appraised to have 1.54 cm rib-fat. Steers were weighed off study the morning of d 141 after 146 DOF and 141 days on study and transported 211 km to a commercial beef abattoir that afternoon to be harvested the following morning (February 13, 2024). Trained individuals entered the slaughter facility, and individual visual identification tags were recorded, RFID tags were recorded via Allflex RS420NFC Series Stick Reader (Allflex USA LLC, Rahway, NJ), and packer identification tags were recorded to ensure individual carcasses could be traced to live steers. Livers were also evaluated for health according to the Elanco Liver Check Service (Elanco U.S., Inc., Greenfield, IN). Following chilling, all carcasses were ribbed for USDA-AMS grading, and quality and yield grade attributes were obtained with camera grading. Dressing percentage was calculated as HCW/Final SBW. Estimated empty body fat (EBF) percentage, empty body weight (EBW), and BW at 28% EBF (AFBW) were calculated from observed carcass traits (Guiroy et al. 2002).

### Efficiency of dietary NE utilization

Observed dietary net energy (NE) and the ratio of observed-to-expected (O:E) dietary NE was assessed for the cumulative feeding period (d 1 to d 141). Carcass-adjusted growth performance was used to calculate performance-based dietary NE to determine efficiency of dietary NE utilization. The performance-based dietary NE was calculated from energy gain (EG; Mcal/d):


$$EG=0.0557\times {\mathrm{ADG}}^{1.097}\times W^{0.75}$$

where *W* is the mean equivalent shrunk body weight (EQSBW; kg; NASEM 2000) calculated from MeBW and AFBW:


$$\mathrm{EQSBW}=\mathrm{MeBW}\times (\frac{478}{\mathrm{AFBW}})$$

Maintenance energy (EM) was calculated as:


EM=0.077×MBW

Dry matter intake is related to energy requirements and dietary NE for maintenance (NEm; Mcal/kg) according to the following equation:


$$\mathrm{DMI}=(\frac{EG}{(0.877NEm)-0.41})+(\frac{EM}{\mathrm{NEm}})$$

and can be resolved for estimation of dietary NEm by means of the quadratic formula:


$$x=\frac{-b\;\pm \;\sqrt{b^{2}-4ac}}{2c},$$

where *a = *−0.41EM, *b = *0.877EM + 0.41DMI + EG, and *c = *−0.877DMI (Zinn and Shen 1998). Dietary NE for gain (NEg; Mcal/kg) can be calculated as:


NEg=0.877NEm-0.41

(Zinn 1987). Estimated DMI was calculated as


$$\mathrm{Estimated}\;\mathrm{DMI}=(\frac{EM}{\mathrm{NEm}})+(\frac{EG}{\mathrm{NEg}})$$

and was presented as a ratio of estimated DMI to observed DMI. Observed-to-expected NEm and NEg were a ratio of performance-based dietary NE to tabular dietary NE values (Preston 2017) and were included to evaluate cattle efficiency relative to the tabular NE value of the diet. A ratio of < 1 indicates that the calculated NE of the diet based on the observed cattle performance was less than expected based on the tabular NE of the diet. A ratio of > 1 indicates that the calculated NE of the diet based on the observed cattle performance was greater than expected based on the tabular NE of the diet. The maintenance coefficient (MQ; Mcal/kg^0.75^) was determined using the following equation (Zinn 1987):


$$MQ=\frac{\left[(\mathrm{DMI}-(\frac{EG}{\mathrm{NEg}}))\times \mathrm{NEm}\right]}{\mathrm{MBW}}$$

### Statistical analysis

All data was analyzed as a randomized complete block design. Day 22 SBW was used as a covariate because feed restriction began on d 22 and SBW differed between treatments. Growth performance, carcass characteristics, and efficiency of dietary NE utilization were analyzed using the MIXED procedure of SAS 9.4 (SAS Inst. Inc., Cary, NC) with pen as the experimental unit. A fixed effect of treatment and a random effect of block were used in the model analysis. Least square means were generated with the LSMEANS option of PROC MIXED and means were separated using Tukey’s adjustment and denoted different (*P* ≤ 0.05). Quality grade, yield grade, and liver scores were analyzed using the GLIMMIX procedure of SAS 9.4 with pen as the experimental unit using a multinomial distribution. Significance was determined at *P* ≤ 0.05 and tendencies at 0.05 < *P* ≤ 0.10.

## Results

Initial shrunk body weight did not differ between treatments (*P* = 0.17; Table 4). Day 22 SBW was used as a covariate so only arithmetic means are presented for d 22 SBW. Body weight measurements on day 50 and 78 were greatest for ADLIB and least for RES56 and RES86 (*P* < 0.01). Day 108 SBW tended to differ (*P* = 0.09) and d 141 SBW did not differ (*P* = 0.82). As designed, RES56 and RES86 ate less than ADLIB on a weight basis (7.60 and 7.79% less, respectively; *P* < 0.01) and as a percentage of BW and MBW (*P* < 0.01) during the restriction period from d 22 to 77. Average daily gain was also 11.48 and 11.64% less for RES56 and RES86, respectively, relative to ADLIB (*P* < 0.01), but G:F did not differ among treatments (*P* = 0.16). Dry matter intake was 5.41 and 7.74% greater for ADLIB compared to RES56 and RES86, respectively, from d 22 to 107 (*P* < 0.01), while ADG and G:F both tended to be different (*P* ≤ 0.10). From day 78 to 141, DMI on a weight and percentage of BW and MBW did not differ (*P* ≥ 0.53) but ADG and G:F were greatest for the RES56 (16.14 and 15.23% greater, respectively) and RES86 (18.66 and 20.39% greater, respectively) cattle (*P* < 0.01). During the recovery period when all cattle were offered ad libitum feed (d 108 to 141), DMI did not differ (*P* ≥ 0.15). Average daily gain was greatest for RES86 and least for ADLIB with the RES56 not different from either (*P* = 0.04), and G:F tended to differ between treatments (*P* = 0.06). Carcass adjusted final SBW also did not differ among treatments (*P* ≥ 0.73). Cumulatively (d 1 to 141) DMI was 3.67 and 4.09% greater for ADLIB compared to RES56 and RES86, respectively (*P* < 0.01). Carcass adjusted and unadjusted ADG did not differ cumulatively (*P* ≥ 0.63), but G:F was greatest for RES86, least for ADLIB, and RES56 did not differ (*P* ≤ 0.04).

**Table 4:** Growth performance of yearling Black Angus steers during the step-up (d 1 to 21), restriction interim (d 22 to 49 and d 50 to 77), RES56 realimentation and RES86 restriction overlap (d 78 to 107), RES56 realimentation interim and RES86 realimentation (d 108 to 141), RES56 restriction (d 22 to 77), RES86 restriction (d22 to 107), RES56 realimentation (d 78 to 141), cumulative (d 1 to 141), and cumulative carcass adjusted (d1 to 141) periods when cattle were fed ad libitum for the entire feeding period (ADLIB), restricted to 92% of ad libitum for 56 d (d 22 to 77) then offered ad libitum for the remainder (RES56), or restricted to 92% of ad libitum for 86 d (d 22 to 107) then offered ad libitum for the remainder (RES86).

Variable	ADLIB	RES56	RES86	SEM	*P*-value^a^
**d 1 to 21**					
** Initial Shrunk Body Weight, kg**	413	411	412	0.7	0.17
** Dry Matter Intake, kg/d**	10.13	10.23	10.14	0.075	0.68
** Dry Matter Intake, % SBW^c^**	2.35	2.38	2.36	0.017	0.51
** Dry Matter Intake, % MBW^d^**	13.06	13.20	13.08	0.094	0.57
** Average Daily Gain, kg/d**	1.73	1.84	1.78	0.031	0.17
** Gain:Feed**	0.170	0.181	0.175	0.0038	0.31
** d 22 Shrunk Body Weight^e^, kg**	452	445	451		
**d 22 to 49**					
** Dry Matter Intake, kg/d**	13.36^f^	12.25^g^	12.28^g^	0.154	< 0.01
** Dry Matter Intake, % SBW^c^**	2.71^f^	2.53^g^	2.52^g^	0.028	< 0.01
** Dry Matter Intake, % MBW^d^**	15.56^f^	14.47^g^	14.41^g^	0.167	< 0.01
** Average Daily Gain, kg/d**	3.12^f^	2.53^g^	2.79^g^	0.098	< 0.01
** Gain:Feed**	0.233	0.209	0.226	0.0069	0.16
** d 50 Shrunk Body Weight, kg**	537^f^	521^g^	528^g^	2.7	< 0.01
**d 50 to 77**					
** Dry Matter Intake, kg/d**	14.99^f^	14.02^g^	13.94^g^	0.216	< 0.01
** Dry Matter Intake, % SBW^c^**	2.62^f^	2.52^g^	2.49^g^	0.037	< 0.01
** Dry Matter Intake, % MBW^d^**	15.60^f^	14.94^g^	14.76^g^	0.219	< 0.01
** Average Daily Gain, kg/d**	2.62	2.60	2.34	0.106	0.04
** Gain:Feed**	0.175	0.187	0.168	0.0081	0.17
** d 78 Shrunk Body Weight, kg**	611^f^	593^g^	593^f^	3.5	< 0.01
**d 78 to 107**					
** Dry Matter Intake, kg/d**	15.12^f^	14.88^f^	14.01^g^	0.220	< 0.01
** Dry Matter Intake, % SBW^c^**	2.37^f^	2.37^f^	2.24^g^	0.032	< 0.01
** Dry Matter Intake, % MBW^d^**	14.52^f^	14.46^f^	13.66^g^	0.199	< 0.01
** Average Daily Gain, kg/d**	1.92^g^	2.43^f^	2.20^f^	0.071	< 0.01
** Gain:Feed**	0.127^g^	0.164^f^	0.157^f^	0.0041	< 0.01
** d 108 Shrunk Body Weight, kg**	668	666	660	3.4	0.09
**d 108 to 141**					
** Dry Matter Intake, kg/d**	14.62	14.63	15.09	0.247	0.28
** Dry Matter Intake, % SBW^c^**	2.12	2.13	2.20	0.034	0.15
** Dry Matter Intake, % MBW^d^**	13.26	13.27	13.72	0.212	0.18
** Average Daily Gain, kg/d**	1.33^g^	1.41^fg^	1.71^f^	0.098	0.04
** Gain:Feed**	0.091	0.096	0.113	0.0063	0.06
** d 141 Shrunk Body Weight, kg**	712	713	715	4.4	0.82
**d 22 to 77**					
** Dry Matter Intake, kg/d**	14.17^f^	13.14^g^	13.11^g^	0.179	< 0.01
** Dry Matter Intake, % SBW^c^**	2.68^f^	2.52^g^	2.52^g^	0.033	< 0.01
** Dry Matter Intake, % MBW^d^**	15.65^f^	14.70^g^	14.66^g^	0.193	< 0.01
** Average Daily Gain, kg/d**	2.87^f^	2.56^g^	2.56^g^	0.062	< 0.01
** Gain:Feed**	0.202	0.197	0.195	0.0048	0.43
**d 22 to 107**					
** Dry Matter Intake, kg/d**	14.51^f^	13.74^g^	13.43^g^	0.176	< 0.01
** Dry Matter Intake, % SBW^c^**	2.60^f^	2.47^g^	2.43^g^	0.030	< 0.01
** Dry Matter Intake, % MBW^d^**	15.39^f^	14.63^g^	14.33^g^	0.177	< 0.01
** Average Daily Gain, kg/d**	2.54	2.51	2.44	0.040	0.09
** Gain:Feed**	0.176	0.183	0.182	0.0030	0.10
**d 78 to 141**					
** Dry Matter Intake, kg/d**	14.87	14.73	14.59	0.211	0.53
** Dry Matter Intake, % SBW^c^**	2.25	2.27	2.23	0.031	0.72
** Dry Matter Intake, % MBW^d^**	13.91	13.93	13.76	0.190	0.72
** Average Daily Gain, kg/d**	1.61^g^	1.90^f^	1.94^f^	0.049	< 0.01
** Gain:Feed**	0.109^g^	0.127^f^	0.134^f^	0.0032	< 0.01
**d 1 to 141**					
** Dry Matter Intake, kg/d**	13.89^f^	13.39^g^	13.34^g^	0.150	< 0.01
** Dry Matter Intake, % SBW^c^**	2.47^f^	2.40^g^	2.37^g^	0.026	< 0.01
** Dry Matter Intake, % MBW^d^**	14.65^f^	14.19^g^	14.05^g^	0.154	< 0.01
** Average Daily Gain, kg/d**	2.13	2.16	2.17	0.030	0.71
** Gain:Feed**	0.154^g^	0.160^fg^	0.163^f^	0.0029	0.04
**d 1 to 141, Carcass-adjusted^h^**					
** Dry Matter Intake, kg/d**					
** Average Daily Gain, kg/d**	2.12	2.13	2.16	0.031	0.63
** Gain:Feed**	0.153^g^	0.159^fg^	0.162^f^	0.0022	0.01
** d 141 Shrunk Body Weight, kg**	710	710	714	4.6	0.73

a Effect of treatment, d 22 SBW included as covariate.

b A 4% pencil shrink was applied to all BW measures to account for gastrointestinal tract fill.

c Dry matter intake as a percentage of median shrunk body weight for the period.

d Dry matter intake as a percentage of median metabolic body weight for the period.

e Values for SBW at day 22 are arithmetic means. All other variables are least squares means adjusted for SBW at day 22.

f,g Means within a row lacking a common superscript differ *P* ≤ 0.05.

h Carcass adjusted final body weight calculated from HCW/0.625.

During the transition period when steers were being transitioned to the final diet, DMI, ADG, and G:F did not differ (*P* ≥ 0.17; Table 4). Within the restriction period, DMI was 8.65 and 8.42% greater for ADLIB compared to RES56 and RES86, respectively, from d 22 to 49 (*P* < 0.01) and 6.68 and 7.24% greater for ADLIB compared to RES56 and RES86, respectively from d 50 to 77 (*P* < 0.01). From d 22 to 49 ADG was 20.74 and 11.15% greater for ADLIB compared to RES56 and RES86, respectively (*P* < 0.01), but G:F did not differ (*P* = 0.16). From d 50 to 77 G:F did not differ (*P* = 0.17), but ADG did differ between treatments (*P* = 0.04). Due to using a Tukey’s mean separation adjustment, treatment means did not differ for ADG from d 50 to 77 despite significance (*P* = 0.04). From d 78 to 107, while RES56 was being offered ad libitum feed but RES86 was still restricted, DMI was 7.63 and 6.01% greater for ADLIB and RES56, respectively, compared to RES86 (*P* < 0.01). Average daily gain was 23.74 and 13.78% greater for RES56 and RES86, respectively, compared to ADLIB (*P* < 0.01), and G:F was 25.42 and 21.49% greater for RES56 and RES86, respectively, compared to ADLIB (*P* < 0.01).

Hot carcass weight, dressing percent, and rib eye area (REA) did not differ between treatments (*P* ≥ 0.27; Table 5). Rib fat thickness and EBF for ADLIB cattle were 9.87 and 3.66% greater, respectively, compared to RES86, with RES56 intermediate and not differing from either (*P* ≤ 0.05). Marbling was 12.97% greater for ADLIB compared to RES56, but RES86 was intermediate and did not differ from either (*P* = 0.02). Empty BW did not differ (*P* = 0.73), but AFBW was 3.86% greater for RES86 relative to ADLIB with RES56 intermediate and not differing (*P* = 0.01).

**Table 5:** Carcass characteristics of yearling Black Angus steers when fed ad libitum for the entire feeding period (ADLIB), restricted to 92% of ad libitum for 56 d (d 22 to 77) then offered ad libitum for the remainder (RES56), or restricted to 92% of ad libitum for 86 d (d 22 to 107) then offered ad libitum for the remainder (RES86).

Variable	ADLIB	RES56	RES86	SEM	*P*-value^a^
**Hot Carcass Weight, kg**	444	444	446	2.9	0.73
**Dressing Percent, %**	62.29	62.28	62.42	0.335	0.94
**Ribeye Area, cm^2^**	88	92	89	1.4	0.27
**Rib Fat, cm**	1.71^b^	1.65^bc^	1.55^c^	0.049	0.05
**Marbling^d^**	620^b^	544^c^	573^bc^	14.2	0.02
**Estimated EBF^e^, %**	33.93^b^	32.75^bc^	32.71^c^	0.285	0.01
**Estimated EBW^f^, kg**	616	616	620	3.8	0.73
**AFBW^g^, kg**	596^c^	617^bc^	619^b^	4.9	0.01
**Yield Grade, %**					0.07
** 2**	6.3	12.6	12.2	–	
** 3**	37.2	49.8	49.3	–	
** 4**	49.7	34.4	35.1	–	
** 5**	6.8	3.3	3.4	–	
**Quality Grade, %**					0.02
** Select**	1.8	4.8	3.6	–	
** Low Choice**	13.5	28.4	23.3	–	
** Average Choice**	28.6	35.1	34.5	–	
** Upper Choice**	35.2	23.0	27.1	–	
** Prime**	20.9	8.7	11.5	–	
**Liver Score^h^, %**					0.49
** Edible**	89.8	88.7	94.3	–	
** Minor**	5.1	5.6	2.9	–	
** Major**	5.1	5.7	2.8	–	

a d 22 SBW included as a covariate in the model.

b,c Means within a row lacking a common superscript differ *P* ≤ 0.05.

d 400 = Small00.

e Estimated percentage empty body fat calculated from Guiroy et al. (2002) = 17.76207 + (4.68142 × fat thickness, cm) + (0.01945 × hot carcass weight, kg + (0.81855 × QG) – (0.06754 × ribeye area, cm^2^); QG = quality grade (Select = 4, Low Choice = 5, Average Choice = 6, High Choice = 7, Low Prime = 8).

f Estimated empty body weight was calculated from Guiroy et al. (2002) = (1.316 × hot carcass weight, kg) + 32.29.

g Final body weight adjusted to 28% empty body weight (Guiroy et al. 2002).

h Evaluated according to the scoring system of Brown and Lawrence (2010): Edible (no abscesses); Minor = A- (1 or 2 small abscesses or inactive scars), A (1 to 2 large abscesses or multiple small abscesses); Major = A+ (multiple large abscesses), A + Adhesion (liver adhered to gastrointestinal tract or diaphragm or both), or A + Open (open liver abscess).

Yield grade distribution tended to be different between treatments (*P* = 0.07; Table 5). Numerically, the ADLIB group had fewer Yield Grades 2 and 3 and more 4 and 5 than the RES56 or RES86. Quality grade distribution was also different between the groups (*P* = 0.02) with the distribution shifting towards Low and Average Choice and a lower proportion towards Upper Choice and Prime grades in the restricted treatments compared to ADLIB. Liver score distribution did not differ among treatments (*P* = 0.49).

Observed NEm and NEg were greatest for RES86 and least for ADLIB with RES56 intermediate and not differing from either (*P* ≤ 0.05; Table 6). Inversely, the MQ was greatest for ADLIB and least for RES86, with RES56 intermediate and not differing (*P* = 0.03). Observed-to-expected ratios of NEm and NEg followed the same trends, with the greatest for RES86 and least for ADLIB, with RES56 intermediate and not differing (*P* ≤ 0.05). The observed-to-expected ratio of DMI was greatest for ADLIB and least for RES86, with the RES56 and RES86 groups not differing from each other (*P* = 0.01).

**Table 6:** Energetics of yearling Black Angus steers when fed ad libitum for the entire feeding period (ADLIB), restricted to 92% of ad libitum for 56 d (d 22 to 77) then offered ad libitum for the remainder (RES56), or restricted to 92% of ad libitum for 86 d (d 22 to 107) then offered ad libitum for the remainder (RES86).

Variable	ADLIB	RES56	RES86	SEM	*P*-value^a^
**Performance Calculated Dietary Net Energy**					
** Maintenance, Mcal/kg**	1.97^b^	2.02^bc^	2.04^c^	0.018	0.04
** Gain, Mcal/kg**	1.33^b^	1.36^bc^	1.38^c^	0.016	0.04
** MQ^d^, Mcal/BW^0.75^**	0.084^c^	0.078^bc^	0.075^b^	0.0026	0.03
**Observed:Expected Ratio**					
** Net Energy for Maintenance**	0.97^b^	0.99^bc^	1.00^c^	0.009	0.04
** Net Energy for Gain**	0.98^b^	1.01^bc^	1.02^c^	0.012	0.04
** Dry Matter Intake**	1.04^c^	1.01^bc^	1.00^b^	0.010	0.01

a d 22 SBW included as a covariate in the model.

b,c Means within a row lacking a common superscript differ *P* ≤ 0.05.

d MQ = Calculated maintenance coefficient: (((Dry matter intake—feed required for gain) × (dietary net energy for maintenance))/(median shrunk body weight)^0.75^).

## Discussion

Cumulatively, both restricted groups had no difference in ADG but decreased DMI relative to the ADLIB group. This resulted in the RES86 group having improved feed efficiency compared to the ADLIB group with the RES56 not differing from either group. Cumulative responses are the primary variables of interest; however, examining interim period responses can explain much of the final outcomes in the restricted cattle. Two primary periods of the study will be discussed, the restriction period when the restricted groups were feed restricted, and the realimentation period, when the restricted groups were offered ad libitum feed after restriction. During the restriction period, DMI was reduced by 8% as designed. ADG was reduced to a greater extent than the feed restriction for the RES56 but not the RES86 compared to ADLIB (12.1 and 4.2%, respectively). Feed efficiency did not differ between the restricted groups and ADLIB during their respective restriction periods.

Within the restriction period, ADG was reduced during the first 28 d (d 22 to 49) for both RES56 and RES86 relative to ADLIB. However, during the subsequent interim period from d 50 to 77, ADG did not differ from ADLIB for RES56 while the RES86 remained depressed. Since both restricted treatments have been under identical restriction conditions up to d 56 of the restriction (d 77 of the experiment), it is unexpected that they have differing responses. These differences in the interim ADG likely reflect differences in weighing variation and gut fill, rather than a physiological divergence, as the cumulative growth performance did not differ between the RES56 and RES86. Regardless, for the final 28 d of the restriction period, the restricted groups match or even outperform ADLIB in ADG. This results in no difference in feed efficiency for the first 56 d of restriction, but the last 30 d of restriction for RES86 has improved feed efficiency compared to ADLIB and similar feed efficiency to RES56 over their first 30 d of realimentation. A delay in feed efficiency improvements later in the restriction period has previously been observed by Hicks et al. (1990). Hicks et al. (1990) observed that steers limit-fed 85% of ad libitum did not differ in feed efficiency from the ad libitum fed cattle for the first 56 d but had improved feed efficiency during the final 84 d and noted similar results in heifers as well. Improvements in feed efficiency may be caused by decreases in gastro-intestinal tract and visceral organ size, which may take a while to respond to decreases in feed intake resulting in a delay in these improvements.

The realimentation period is when the greatest growth benefits of restricted feeding are observed. Both restricted groups saw enhanced growth during the realimentation period because of compensatory growth. Interestingly, this growth was driven not by increasing feed intake to a greater extent than the ADLIB group either as a percentage of BW and MBW or on a weight per day basis, but rather by improved feed efficiency. The increase in feed efficiency is caused by multiple factors, primarily decreased maintenance requirements and changes in the composition of growth (Sainz and Bentley 1997; Galyean et al. 1999). The O:E ratio for NEm was greatest and the maintenance coefficient was least for RES86 over the entire feeding period, supporting the theory that maintenance requirements are decreased for restricted fed cattle.

The composition of gain in cattle is primarily a function of ADG and body weight relative to their mature body weight (Lofgreen and Garrett 1968). By restricting feed intake, cattle growth is slowed and their mature body weight is increased while they are also lighter than their ad libitum fed counterparts. This results in greater protein and reduced fat deposition (Lofgreen and Garrett 1968). Although REA was not affected by restricted feeding, most measures of fat were reduced by restricted feeding, especially the RES86 group. The only measure of fat that the RES86 group was not statistically different from ADLIB was marbling, however RES86 cattle had 8% lower marbling scores than ADLIB and the quality grade distribution for both restricted groups was shifted away from Prime and upper Choice towards more Select and Low and Average Choice. Hicks et al. (1990) observed a shift from 61% to 42% choice carcasses in ad libitum vs. limit-fed steers, and Murphy and Loerch (1994) also noted steers grading Choice decreased from 100% when offered ad libitum feed to 67% and 33% when steers were offered 90% and 80% ad libitum feed, respectively. A long realimentation period may help minimize the decrease in intramuscular fat (Loerch and Fluharty 1998). When limit fed cattle for at least 96d were subsequently offered feed ad libitum, no differences in carcass characteristics were noted (Loerch and Fluharty 1998).

As a result of the decreased fat deposition, restricted cattle had a greater AFBW, which is the BW at which cattle reach 28% EBF, or indicate when cattle grade Choice. A greater AFBW suggests an animal will be heavier at 28% EBF and can be finished to a heavier FSBW at a similar EBF. This compounded the decreased fat deposition as the restricted cattle also had a lighter BW relative to their mature BW. The difference in AFBW suggests that if cattle were fed to the same degree of fatness (28% EBF), the RES56 and RES86 cattle would have 21 and 23 kg heavier final BW, respectively. Although not measured directly, because of the decrease in intramuscular and subcutaneous fat with no difference in HCW or REA, we hypothesized that the restricted cattle had a greater proportion of protein and less fat in their carcass composition. Calculated EBF was also least for RES86. In 90% and 80% feed restricted steers, carcass ether-extractable lipids were reduced 10.2% and 25.1% and carcass protein was increased 2.6% and 7.9% compared to the ad libitum group, respectively (Murphy and Loerch 1994).

Although cattle reached similar final BW and HCW, both restricted groups had decreased rib fat thickness and marbling. As previous research has shown (Murphy and Loerch 1994; Galyean et al. 1999), increasing the realimentation period can help alleviate this effect. The primary limitation of this experiment was cattle were all harvested at the same days on feed, which was necessary to determine the effects of the limit feeding period relative to ad libitum feeding. Further research is needed to determine the length the realimentation period needs to be to reach similar fatness and carcass characteristics to ad libitum cattle.

## Conclusion

Restricting feed intake by 8% of ad libitum before returning cattle to ad libitum feed provides an improvement in feed efficiency with no effect on live performance, however, marbling and carcass quality are reduced. The primary drivers of the increased feed efficiency are likely decreased maintenance requirements during the realimentation period, as well as a shift in composition of gain towards more lean tissue and less fat. Greater DOF, specifically during the realimentation period, may help alleviate some of the decreases in carcass quality but will likely remove the reductions in feed intake and improved feed efficiency that we observed in this study.

## Data Availability

Data can be made available upon reasonable request to ZKS.

## List of abbreviations

ADF: acid detergent fiber
ADG: average daily gain
AFBW: estimated body weight at 28% empty body fat
CP: crude protein
DM: dry matter
DMI: dry matter intake
DOF: days on feed
EBF: empty body fat
EBW: empty body weight
EDDI: ethylenediamine dihydroiodide
EE: ether extract
EG: daily energy gain
EM: maintenance energy required
EQSBW: equivalent shrunk body weight
G:F: gain to feed ratio
HCW: hot carcass weight
MBW: median feeding shrunk body weight
MeBW: median shrunk body weight
MDGS: modified distillers plus solubles
MQ: maintenance coefficient
NDF: neutral detergent fiber
NE: net energy
NEg: net energy for gain
NEm: net energy for maintenance
O:E: observed to expected
REA: ribeye area
RFID: radio-frequency identification
SBW: shrunk body weight
